# Chicago Medical Society

**Published:** 1889-03

**Authors:** 


### SOCIETY REPORTS.

### CHICAGO MEDICAL SOCIETY.

*Regular Meeting December 27, 1888.*

The President in the Chair.

Dr. J. R. Brandt read a paper on

DIPHTHERIA MALIGNA.

He said: When I state that in 1881 in Buda Pesth there was an epidemic during which 43,000 persons were attacked, and 19,000 died, nearly 45 per cent., none recovering under fifteen years of age; and that in 1883, in New York city, there were 3,502 cases and 2,090 deaths, or over 59 per cent., we must conclude that the study of this disease alone is of sufficient importance to require and demand the fullest attention of the medical profession, and we should search continually for a prophylactic or a remedy.

In regard to diagnosis, it may seem as though little is required to be said, as the diagnosis is frequently made by the laity before the physician is called. Some eight years ago I thought I had made a discovery of several prodromatic signs in diphtheria. Since then I have gone on making careful investigations, the results of which prove that I was correct in my impressions. At that time I made note of my alleged discoveries and published a short notice in their regard in the *Medical Record*, of which the following is a copy: "In February, 1881, Dr. J. R. Brandt, of Robinson, Wis., writes: I wish to state for the benefit of the profession that in examining members of families suffering from diphtheria I have found in several of those not afflicted a notable fall of temperature, from half a degree to a degree and a half, and a noticeable slowness of pulse, irregularity of the heart's action, loss of beat every 3″, 5″, 7″, or 3″, 9″, 14″, from 12 to 36 hours before there was any other exhibition of the disease—such cases I have found to be malignant." My rule is to carefully examine each member seemingly unaffected, as to temperature, action of heart, pulse, and complexion of fauces. I leave a thermometer with a well-instructed person with orders to take temperature every hour, night and day, either in groin, axilla, mouth or rectum, and keep a record of same until I come again, always making two or three visits a day, even if I have to neglect other work. Then when I find two out of three of these symptoms exhibited I begin treatment at once. I have since 1886 had 149 cases which exhibited these symptoms, and I have been able to save all but one, which case was tubercular.

In regard to surgical therapeutics, caustics, such as argenti nitras or hydrochloric acid fortier are only mentioned to be condemned. Blistering the neck and chest by using any vesicants as counter irritation only give a larger surface for developing an exudate and poisoning the system. Sprays in my hands have not given satisfaction that others claim. I have seen several cases of broncho-pneumonia induced by the use of the spray; even an antiseptic spray softens the membrane and small particles are carried by inhalation into the air vesicles, plugging them and establishing centers for spreading the disease, and these particles also produce mechanical obstruction and prevent oxidation of the blood. Other particles are swallowed and interfere with digestion. They also may be absorbed and carried into the circulation. The same reasoning is eminently true of inhalants. Irrigation when applied to the nasal cavities is of incalculable advantage, as nasal diphtheria is very fatal on account of the large surface attacked and their lymph communication with the rest of the body. (Jacobi) I take a household syringe and attach it to a soft, hollow bougie and introduce it into the nasal cavity, horizontally if the patient is sitting, and perpendicularly if lying in the nurse's lap, gently compressing the bulb and turning the bougie so that the stream from it will be applied to different parts with sufficient force to dislodge portions of the membrane and force them out and bathe the surface with antiseptic fluid. I like this better than the rubber open at the end, by using which you may force particles down the throat which should be avoided. I use for this purpose bichlo. sol. 1-5000, warm, and every three and four hours, or even every hour for a time, endeavoring to keep an open passage. I use at one time from 4 to 6 ozs. in each cavity; nothing will reduce adenitis as irrigation. Gargles have never in my hands been of any advantage and I gave them up long ago.

I have never seen a patient recover on whom tracheotomy had been performed after crepitant and subcrepitant râles had developed at the base of the lungs. If not allowed to operate before this condition arises I decline to operate, for we only prolong the sufferings of the patient. I believe tracheotomy should be prophylactic, the same as in medical therapeutics. Throw the responsibility on the relatives and friends and give a very guarded prognosis, even when the operation is done early. As to intubation, much is now being said in its favor, and in feeble and young patients it is giving satisfaction, but in malignant cases, such as this article refers to, I prefer tracheotomy. In intubation portions of false membrane may be forced down the trachea, or the tube come in contact with the membrane and hold in situ, where it will be absorbed; the tube, by pressure, may cut off circulation at that point and increase the putrefying surface. Should the tube become plugged from below, there is danger of suffocation before an expert might be obtained to remove it, as in tracheotomy an expert can remove the canula at once. In cases (as Dr. Parker, of London, has pointed out) in which the dry epiglottic folds have become tumefied, it is impossible to introduce the tube. Admitting this to be awkwardness, it only shows that time and practice will have to be taken to make one an expert, for expertness is most certainly required to make this procedure a success.

Dr. Waxham has reported 160 cases with 44 recoveries, making 28 per cent. of recoveries, of 1,072 tracheotomies performed in various parts of the United States. There have been 287 recoveries, or 27 per cent., which look favorable for tubage, but I think before giving statistics we must have more intubation. I believe the better way would be to intubate in non-malignants, and to perform tracheotomy in malignants; always intubate early or not at all.

In the treatment of patients with diphtheria, there are several hygienic rules I strictly enforce: first sequestration if it can possibly be done. In farming districts I utilize the barns, smoke-houses and granaries, in winter; in summer and fall I improvise tents. I give them earthen floors if possible, I never allow the wooden floors to be washed, but have them sprinkled with a bichloride solution every six hours, 1-1000. Plenty of fresh air constantly passing through the rooms, the drinkable water always boiled, and afterward cooled by surrounding it with ice in vessels. Milk I order boiled and treated in the same way. I never allow the patient to take one step under any circumstance; they must always be carried or handled in such a manner that they will make no exertion. I make from three to six visits each day, personally and carefully examining their throats, using a head mirror at night and always wearing glasses to protect my eyes. With malignants I have remained for seventy-two consecutive hours. I will not leave a case until some suitable person can be obtained and whom I have personally instructed. During the epidemic in Wisconsin, which lasted from 1876 to 1880, I used every remedy which gave any reason for success, mentioned in the current medical literature of the day, and I have found much more to condemn than to approve; so many were worse than worthless that I will give only those which have proved to be of the most value in my hands, and the manner of using them. I advise general and local treatment, as I consider it a systemic disease with local manifestations. The use of alkalines was very unsatisfactory, but the reason is patent when we reflect that putrefactive changes take place with great rapidity in alkaline albuminoids, and less when there is an acid. Acid retards putrefactive changes, and so does alcohol, and herein lies the reasons for rational treatment. In malignant cases I give acids to antagonize the changes going on and producing ptomaines; I give alcoholic stimulants which also retard putrefaction and degeneration, and give food, best milk, or milk and coffee, cream and coffee, anoint with cream and whisky the whole body every six hours. Give rectal injection of the most easily digested foods to make new blood to supply the economy which is below par. The ptomaine may be eliminated or oxidized. The inhalation of oxygen is worthy of trial where it can be done, and I have been pleased with its effects; it acts both as a destroyer of the ptomaines and retards putrefaction. I use tr. ferri and the bichlor. hydrag. after the following formula for an adult:

Tr. chlor. iron..............ʒ iv.
Bichlor. mercury..........gr. i.
Syrup simple to...........℥ iv.
Sig. One teaspoonful every two hours.

Alternate with

Hydrochloric acid dil......ʒ iv.

Com. tr. cinchon..........℥ iss.

Syr. sim. to...............℥ iv.

Sig. Teaspoonful every two hours.

In a few moments after each I give whisky mixed with water and sugar from 1 to 3 teaspoonfuls, in a moment after I give ¼ of a teacupful of milk that has been boiled and cooled in ice. This treatment I continue for from 24 to 72 hours, depending upon the gravity of the case, when I give a little more time, *i. e.* one hour and a half for 24 or 36 hours, and if all is doing well I then alternate every two hours until recovery. When I find an exudate on the tonsils I touch it with a solution of subsulphate of iron, one half, and bichloride of mercury, one-half, 1 to 250, using a camel's hair brush; applying this preparation every two hours just previous to administering the iron mixture; immediately I remove all the shreds of membrane I can, even using dissecting forceps for the purpose; then on this surface I again apply the above antiseptic solution. I have greater dread of the superimposed layers of putrefying membrane being absorbed and completely saturating the system, and producing more ptomaines, than I have of doing harm by more or less irritating these parts. All agree that the removal of the putrefying membrane from the nasal cavities by irrigation is good treatment; if good for one why not for another part? In malignant cases that do recover the patients should not be allowed to leave their bed for three or four weeks, and watched carefully for heart failure. Malignant diphtheria in tuberculous subjects is nearly always fatal. Malignants develop mild forms in others and the converse; these deductions I have derived from, and after having treated and recorded in the last twelve years 2,600 cases.

There are certain precautions necessary to be taken by the physician, and first I deem it criminal for a physician to attend cases of confinement while in attendance upon either scarlatina, diphtheria or erysipelas. As I have found dogs and rabbits infected with diphtheria, I think all pets should be removed from the house at once, as the more pets the more sources of infection. In all cases of nursing infants, to medicate the mother is to save the child, that is, if the child will take the breast, and they will take the breast in most cases where they will refuse every thing else. Powders have not been satisfactory except when applied to the exudate found on the genitialia of females, and here calomel and tannic acid has been of great benefit. Pilocarpine has been followed by such extreme exhaustion that I deem it too hazardous to risk. In heart failure I depend upon alcoholic stimulants and rest, not allowing the patient to make any exertion whatever. Digitalis is so poorly borne by the stomach that I give it *per rectum* or hypodermically; I never use digitaline. Camphor and carbolic acid in equal parts applied to the exudate was not as satisfactory as subsulphate of iron and bichloride of mercury. I have shortened the course of many cases by giving one full dose of calomel at the time of my first visit.

Dr. F. E. Waxham: I have listened with pleasure and profit to this valuable paper, but cannot agree with the author in all his conclusions. The title of the paper is "Diphtheria Maligna." It is often difficult to draw the line between malignant and non-malignant diphtheria, to state where one leaves off and the other begins. I make three classifications of the disease, the mild, the semi-malignant, and the malignant, styling those cases malignant that are characterized by alarming symptoms, by great depression and by a rapidly fatal termination. I believe that those malignant cases that recover are very few and far between. The line of treatment I have found most useful in the management of malignant cases has been the frequent use of bichloride of mercury, alternated with tincture of iron and glycerine, the use of a gargle of bichloride of mercury in dilute alcohol, or, where the patient cannot gargle, the use of the same solution, by the atomizer; free stimulation, abundant nourishment and appropriate treatment of the nasal cavities. Under this treatment I have seen semi-malignant cases, that promised rapid development into malignancy, yield and gradually recover. The fault I usually find is that the medicines are not given with sufficient frequency. The bichloride of mercury should be given at least every hour, alternating with the tincture of iron, so that the throat will be bathed at least every half hour with these solutions. I do not believe that it is best to harrass the patient by the frequent application of solvents to the diphtheritic membrane, for I have learned not to fear diphtheritic membrane as long as it does not invade the larynx and is frequently bathed with antiseptic solutions.

In regard to the surgical treatment of malignant diphtheria, we may well say that it is of little or no avail. In the semi-malignant forms, intubation will save many, but in the very malignant forms of diphtheria I believe that intubation, tracheotomy or any other surgical measure is entirely useless as far as saving life is concerned. I remember two years ago I attempted to perform tracheotomy in a case of malignant diphtheria in which the larynx had become invaded. Almost with the first breath of the anæsthetic, the respiration ceased and the child was dead before the first incision was made.

I should, perhaps, say a word in regard to the prevention of malignant diphtheria. Inasmuch as the medicinal and surgical treatment of malignant diphtheria is discouraging, the preventive treatment is of corresponding importance. It, however, is oftentimes a difficult problem to know how we shall prevent the spread of malignant diphtheria, especially when it occurs in the homes of the poor and among the overcrowded. We will frequently be called to a case of malignant diphtheria, where the child, coming down with the disease, is surrounded with six or eight other children, and we will usually find them crowded in two or three small rooms, and usually two or three of the well children will be sharing the bed at night with the patient.

It becomes impossible to isolate these patients on account of their crowded condition, and very seldom indeed are the friends able to send the healthy children away from home, because they have no friends or relatives who will take them, and there is in this city no hospital where poor patients will be received when suffering from diphtheria. True, if patients have an abundance of means, they can secure a private room in any of our general hospitals, but poor people, who have not means, find it impossible to gain admission to any of our hospitals in Chicago. I have been informed that a young woman with diphtheria recently made application to our County Hospital, but she found it impossible to gain admission—and sick, destitute and penniless she was turned out upon the street.

I hope the time is not far distant when our commissioners will establish a pavilion for infectious diseases, or, this failing, I hope a hospital will be established in Chicago for the exclusive use of these diphtheritic patients. If we send the first patient that comes down with diphtheria to such a hospital, we will frequently be able to prevent the other children from contracting the disease. Indeed, I believe if we had such a hospital, where patients could secure careful nursing and skillful treatment, that it would be overcrowded, and we should be successful in saving many patients and very greatly lessening the mortality from this disease.

Dr. J. H. Chew read a paper entitled

DEVENTER'S METHOD OF DELIVERING THE AFTER-COMING HEAD.

A year or more ago a member of this society, while "pondering over many a quaint and curious volume of forgotten lore," discovered the lost art of delivering the after-coming head by the method practiced and referred to by Deventer, over 160 years ago. In a paper read before the International Medical Congress in Washington, Dr. Bartlett described this as differing from the usual method in the following points: As soon as the child has passed so far as the base of the thorax, extractive efforts are to be made, the woman resting on her back, not in the line of the body of the parturient, but in a direction perpendicular to the floor. When the arms are within reach we are not to stop and draw them down, a thing often difficult to perform and requiring much time; but to examine and see that they are extended by the side of the head and in front of the parietal protuberances. We are then to make forcible traction in the same direction, at the same time urging the mother to bear down with all her power, and if necessary, we are to make pressure upon the occipital end of the head as nearly behind the anterior wall of the pelvis as practicable. The occiput appears under the pubes and the delivery is accomplished with the head in forcible extension instead of flexion. I have been fortunate in delivering by this method, and thereby, I believe, saved the life of the child and spared the mother many minutes of suffering.

I was called the night of October 3, to attend Mrs. S. in labor at eight months: The child presented by both feet, and the labor progressed satisfactorily until the shoulders were delivered—the arms were delivered with the shoulders. I endeavored to deliver in the usual way, but in trying to reach the chin found it high up and the head extended. We immediately placed the woman upon her side and I made traction directly backward, at a right angle to the axis of the mother's body, and then when the occiput appeared under the pubes backward and slightly upward towards the mother's head, at the same time Dr. Wadsworth, who assisted me in the case, made pressure on the head just back of the pubes; in a few moments we had the pleasure of seeing the occiput glide from under the pubes and the delivery accomplished; and to my gratification and surprise—for I feared the traction had been carried to a dangerous degree—the child almost immediately cried out. The child was of average size and has flourished since, although bottle-fed. The mother's recovery from the labor was uninterrupted, although under the most adverse circumstances.

Dr. John Bartlett: The case presented this evening by Dr. Chew and a similar one occurring in the practice of Dr. Wadsworth, are the only ones which have come to my knowledge within the year. It would seem at first glance surprising that experience in this mode of delivering the after-coming head is not gained more rapidly. A little reflection, however, will make it plain why Deventer's method is not more often put to the test. The whole tendency of the teaching regarding the management of head-last labors has been to mould the practice of the operator into a line of action the reverse of the one in question. The practitioner has been taught that one of the chief ills which may overtake an accoucheur in footling cases arises from the departure of the chin from the breast, the extension of the head. From education, and even from habit, his aim has been to prevent this departure of the chin and to secure the maintenance of the flexed attitude of the head. In the majority of the head-last cases occurring to experienced practitioners there is a fairly confident assurance that delivery will occur, or can be effected safely to the child, by the ordinary mechanism and methods. Under these circumstances the adoption *ab initio* of Deventer's plan savors too much of experimentation; so that the conservative accoucheur, recognizing his obligation to his patients as paramount to questions of scientific obstetrical interest, delivers in the old way at the sacrifice of experience in the new method.

In ordinary cases, at least when the Deventer method is practiced in the cautious manner which the obstetrician would at first naturally adopt, it will be found that the extension of the chin will take place only so far as to lock the head in the pelvis so tightly as to lead the operator to desist from his purpose of delivering by the new plan. This want of success is not always due to difficulties inherent in the method. In good part it may be ascribed to the imperfect or inefficient practice of the plan.

The errors usually committed in attempting to deliver by the method of Deventer are: failure promptly to decide upon using that method and consequent delay in drawing the child downward; failure to regard Deventer's caution not to bring down the arms; failure to direct properly the traction, which is generally made directly downward, when it should be downward and slightly backward; failure to make sufficient extractive effort; failure to sway the body of the child from side to side; failure to secure the coöperation of the patient, by urging her to assist with all her power; failure to exert proper auxiliary pressure downward upon the head.

What, it will be inquired, is the result in the event of one's having tried the Deventer method and failed therein? Naturally the inexperienced in these efforts would have misgivings lest the second condition of affairs should be worse than the first. One might naturally suppose that the head would be found locked in the pelvis in the hitherto much dreaded attitude of extension. Such is not the case. According to my limited experience it is because the head is not in extension that delivery by the new method has failed. So far, neither my neighbors nor myself have in anywise added to the difficulties of the case by having made an unsuccessful effort to deliver by the new method. In every instance, on the contrary, the delivery appears to have been advanced by the unsuccessful effort to end the labor by extension. The tractions made have brought the child lower, so that embarrassments as regards the arms have been lessened. Thus, in one case mentioned by Dr. J. W. Niles: whereas, prior to the traction, the doctor had been unable to bring down the arms, these fell down during his unsuccessful effort at delivery after the Deventer plan. And again, after such attempts, the head is found lower in the pelvis, especially as regards the occiput. Now, the end of the head-ovoid having been thus advanced apace, in our experience, the subsequent advance of the opposite extremity, the chin, by the old methods, has been decidedly facilitated.

The question has been asked whether in the present state of our knowledge of this method the practitioner be justified in attempting it in every case? Possibly a perfect knowledge of Deventer's method may finally establish it as the preferable mode of delivery; but in our present inexperience the question just put must be answered negatively. The method may be resorted to when the child is small, or when, as sometimes happens after turning, or in natural labor, as in the cases reported this evening, there is difficulty in delivering in the usual way, whether because the arms can not be brought down, or especially because the chin has departed from the chest in such a manner as to offer an obstacle to the ordinary procedure. A knowledge of the Deventer method even in the developmental state in which it now seems to be, proves a satisfaction to the obstetrician in attendance on a head-last case, for, should difficulty arise with the arms, he can draw the body down in the Deventer line with the confidence of bringing them within reach and delivering them, or, he can feel reassured, the chin being upward, in leaving them to be delivered alongside of the head. Formerly if the chin was found departed from the chest and "hooked up," as the expression was, over the brim of the pelvis, the practitioner regarded the situation as most serious, and even where unusual skill and experience enabled the operator to reverse the position of the head, or, failing in this, to successfully apply the forceps, the child was generally lost by delay. Instead of resorting to the older unsatisfactory method in such cases, he may now draw downward in the Deventer line, with good hope that the head will "shoot through easily."

Certain peculiarities of the pelvis, or of the head, may have a tendency to increase the difficulty of delivering by this method. In a multipara in the practice of Dr. J. M. Hall, in which the pelvis was large and the child small, everything promised success in the employment of this plan. Dr. Hall attempted the delivery exactly *secundem artem*, and yet the chin appeared before the occiput. In this case the head was hydrocephalic, large, round and soft, a condition which it is not to be doubted antagonized the efforts to secure a Deventer delivery.

It may be well here to caution the practitioner against too forcibly pressing the thorax of the child against the perineum, lest internal injury be caused thereby. Against the use of too great extractive force it is hardly necessary to give a caution. And yet the practitioner should have a knowledge of such facts, as the experience of Goodell and others teaches regarding what may be deemed an allowable degree of traction.

Smellie, in referring to Deventer's method, speaks of the head turning in the pelvis, but possibly the first step in this method may occur before the head enters the pelvis; namely, the catching of the chin on the pelvic brim, and the descent of the occiput in advance of it.

The more I see of the attempts to deliver by Deventer's method the more I incline to the opinion that in this form of delivery, as in head-first cases, the *sine qua non* of success is the early descent of the occiput. There is reason to believe that there was something in the manner of the execution of his method by Deventer which led to this early separation of the chin from the breast. Possibly the traction on the body of the child, recommended by Deventer, "so soon as it is out above half way," was so directed as to invite the very end in question, the arrest of the chin and the descent of the occiput in advance of it.

Hitherto in our experience, when the head has been locked in the pelvis from unsuccessful extractive efforts, its long diameters have been found resting in the conjugate diameter. Now, nothing would seem to be plainer than that the turning of the head on its long axis could take place more readily in an oblique diameter than in the antero-posterior one. In the one case, the surfaces resisting the extremities of the lever in our effort at extraction are the posterior face of the body of the pubic bones, and the anterior surface of the sacrum. In the other case they are the soft tissues overlying osseous lacunæ, the foramina ovalia, and the great sacro sciatic notches.

Dr. J. S. Knox: It strikes me, in listening to the paper and its arguments, that the purpose is to convince us that the best method of delivering the after-coming head, is by backward traction; causing extension of the chin, the upward passage of the arms, and descent of the occiput. It is beyond question that, with the chin flexed on the chest, the chin descending first, and the occiput last, the head rotating on the symphysis pubes, the child presents the smallest diameters and the most favorable engagement. Deventer's method, to excel, must present smaller cephalic diameters, and a more favorable adjustment to the maternal passage. I believe this to be impossible. It is a mistaken idea that in Deventer's method the chin catches at the superior strait and the occiput descends and glides under the pubic arch. It is a mechanical impossibility. The distance from the extended chin to the sternum is about two and a half inches, and from the promontory of the sacrum to the point of the coccyx over five inches. Of necessity then the occiput and chest of the child must engage in the pelvis together, in order to deliver the occiput. This is impossible.

In Deventer's method, therefore, the after-coming head must pass through the superior strait, in a flexed position, until the throat of the child reaches the perineum. Backward traction will then fix the chin and bring down the occiput, accomplishing its delivery under the pubic arch. To do this, however, the longest diameter of the head, the occipito-mental, must sweep through the oblique diameter of the maternal pelvis. What is the advantage in substituting a cephalic diameter of $5\frac{1}{4}$ inches for one of $3\frac{3}{4}$? Deventer's method undoubtedly has its place. In those cases where injudicious traction has been made on the body, and the chin has extended, and the arms passed up, this method will afford a much easier delivery of the head. I have carefully studied Dr. Bartlett's paper presented to the International Congress, and have a high regard for his obstetric opinions. But why we should provoke a malposition, and then hunt for a new method of delivery, I cannot see.

Dr. Frank Cary: Dr. Schwandt has tried this method in my service in St. Luke's hospital, and I was very favorably impressed with it.

Dr. E. J. Schwandt: The two methods, I think, are quite different. The method referred to by Dr. Cary was in two or three breech presentations which occurred while I was serving in the obstetrical department of the hospital. Before this method became generally known I did as I was taught in my earlier days, I maintained firm pressure on the fundus uteri and thus prevented extension or departure of the chin from the chest and unfolding of the arms. I therefore concluded to deliver, in the next breech presentation, by the method now under discussion; taking the body of the child, the arms being unborn, and drawing it strongly backwards so that the abdomen of the child came in contact with the buttocks of the mother, paying no attention whatever to extension of the head and unfolding of the arms. In that way I noticed the occiput being born with the chin and arms extended—the occiput slipping out under the symphysis pubes. Furthermore the perinæum is not usually ruptured as one might suppose by this method and birth is easy.

Dr. Rosa H. Engert: This is the first time that I have heard of Dr. Bartlett's method, but I am very much pleased to find that it is the one that I have employed as long as I have been in practice. Once I did not succeed in getting the child alive and after that I practiced on the phantom to find a better way of getting the fœtus out, and found that it was the most natural way to draw the body of the fœtus toward the back of the woman, she being in a side position, the head then passing through very easily, in the smallest diameter, with the occiput coming out first from under the pubes.

Dr. John Bartlett: If Deventer's statements are to be believed, and Smellie's interpretation of that author's plan is to be accepted, delivery with the occiput foremost "sometimes succeeds better than the other method." Dr. Knox's success in delivering living children in head-last labors, by the accepted modes, is certainly very exceptional. According to statistics cited by Zweifel, in 3,475 versions the mortality to the child was nearly 59 per cent. I have taken a very conservative position in the matter of employing the "new" method. I am not convinced that Deventer's plan is yet fully understood. There is, however, certainly enough of evidence in favor of the method as described by Smellie to warrant us in trying it in proper cases.

There is one point in the method of delivery according to Deventer, which has not been touched upon here, and which should be duly considered because it is one of those mechanical conditions in which this plan seems to offer a great advantage over the accepted modes of delivery. When we try to extract the head by ordinary modes, the arms being extended alongside of it, our efforts tend to flex the head and to force it as a wedge more and more firmly between the arms, which thus *key* it in the bony ring of the inferior strait. In the Deventer plan, on the contrary, the movement of extension releases the wedge from between the arms, these slipping more and more over the face of the child, so that the head is delivered before them. I call your attention to the further fact, that while in the ordinary method of delivery the extended arms are crowded towards osseous tissue, in the plan under discussion they are pressed against less unyielding structures.

